# The genetics of obesity: *FTO* leads the way

**DOI:** 10.1016/j.tig.2010.02.006

**Published:** 2010-06

**Authors:** Katherine A. Fawcett, Inês Barroso

**Affiliations:** 1 Metabolic Disease Group, Wellcome Trust Sanger Institute, Wellcome Trust Genome Campus, Hinxton, Cambridgeshire, CB10 1SA, UK; 2 University of Cambridge Metabolic Research Laboratories, Institute of Metabolic Science Addenbrooke's Hospital Cambridge CB2 OQQ, UK

## Abstract

In 2007, an association of single nucleotide polymorphisms (SNPs) in the fat mass and obesity-associated (*FTO*) gene region with body mass index (BMI) and risk of obesity was identified in multiple populations, making *FTO* the first locus unequivocally associated with adiposity. At the time, *FTO* was a gene of unknown function and it was not known whether these SNPs exerted their effect on adiposity by affecting *FTO* or neighboring genes. Therefore, this breakthrough association inspired a wealth of *in silico*, *in vitro*, and *in vivo* analyses in model organisms and humans to improve knowledge of *FTO* function. These studies suggested that FTO plays a role in controlling feeding behavior and energy expenditure. Here, we review the approaches taken that provide a blueprint for the study of other obesity-associated genes in the hope that this strategy will result in increased understanding of the biological mechanisms underlying body weight regulation.

## Obesity: a growing problem

Overweight and obesity, defined as body mass indices (BMI) >25 and >30, respectively, are associated with premature death through increased risk of many chronic diseases, including type 2 diabetes, cardiovascular disease and cancer [1]. Over the last three decades, the prevalence of overweight and obesity have increased rapidly and the latest World Health Organization (WHO) estimates suggest that 1.6 billion adults (aged 15 years and over) were overweight and 400 million were obese in 2005. These figures are predicted to rise to 2.3 billion overweight and over 700 million obese adults by 2015 (http://www.who.int/mediacentre/factsheets/fs311/en/index.html). Obesity is therefore a major international public health threat and economic burden. Although environmental factors, such as little physical activity and over-eating, have driven the recent rise in the numbers of people who are overweight or obese, genetic factors are estimated to account for 40–90% of the population variation in BMI [2–4]. It is hoped that identifying the genetic factors underlying the heritable risk of obesity will contribute to our basic knowledge of the biology of energy balance, and might even highlight molecules and pathways that can be targeted for therapeutic intervention. In 2007, single nucleotide polymorphisms (SNPs) within the fat mass and obesity-associated gene (*FTO*) became the first to be associated reproducibly with human body mass. We review the implications of genetic association between SNPs in the *FTO* gene region and BMI in humans, the various studies undertaken and the challenges in progressing from a genetic association to new biological insight. We also provide an overview of possible future directions for research in this field.

## Genetic studies of common obesity

Before 2007, despite huge efforts using genome-wide linkage studies and candidate gene association studies, no genetic variation had been unequivocally associated with BMI and risk of obesity in population studies [5]. In recent years, however, genome-wide association studies (GWAS), which test the correlation between SNPs across the entire genome and trait variation in a sample of individuals, have succeeded in identifying variants associated reproducibly with complex traits. GWAS for type 2 diabetes (T2D) detected strong association between common SNPs in the *FTO* region and risk of T2D [6–8]. However, subsequent analyses showed that the association between *FTO* SNPs and T2D was mediated by an association with BMI [7]. The association between *FTO* SNPs and BMI and the risk of being overweight or obese has been confirmed in multiple populations [6,7,9–27]. The effect of *FTO* SNPs on BMI is modest, with those individuals homozygous for the risk allele weighing, on average, 3 kg more than those homozygous for the protective allele [7].

Association does not necessarily mean causation. BMI-associated SNPs lie within a 47 kilobase (kb) linkage disequilibrium (LD) block encompassing parts of the first two introns as well as exon 2 of *FTO.* Thus, the association signal could be due to correlation between *FTO* intronic SNPs and variation elsewhere in the gene or control elements of other genes. Indeed, the transcription start site of the retinitis pigmentosa GTPase regulator-interacting protein 1-like (*RPGRIP1L*) gene (homolog of murine *Rpgrip1l*, also known as *Ftm*) is in close proximity (∼400 bp) to the 5’ end of *FTO* [7]. It is therefore possible that SNPs in *FTO* are associated with obesity through an effect on *RPGRIP1L.* Efforts to fine-map the association signal to causative variant(s) are underway (Box 1).

Since the discovery of the *FTO* signal, additional GWAS have succeeded in identifying many additional novel obesity loci [12,28–33]. Before these findings can be converted into clinical benefits, however, it is necessary to determine the biological mechanisms by which confirmed obesity susceptibility variants impact BMI.

## Early insights into *FTO* function

When associations between SNPs at *FTO* and BMI were first discovered [6,7,9], little was known about the function of the *FTO* gene product. *Fto* was first identified in the mouse by positional cloning [34] as one of the genes within a 1.6 megabase deletion on chromosome 8 responsible for the Fused toes (Ft) phenotype [35]. Mice homozygous for the deletion died mid-gestation and exhibited severe malformations of the head and face, central nervous system (CNS) developmental defects [36,37], randomized left–right asymmetry [38], polydactyly and growth retardation. Heterozygotes displayed fused toes and enlargement of the thymus [34,35]. However, no obesity or thinness was reported in these mice. Nevertheless, as *Fto* was only one of six genes deleted in the Ft mouse (the others were the Iroquois B cluster of genes, *Irx3*, *Irx5* and *Irx6*, and two other genes, *Fts* and *Rpgrip1l*), it was not known which, if any, of these phenotypic manifestations were due to *Fto* deficiency. Interestingly, a phenotype similar to that of Ft mice was seen in a human patient harboring a small chromosomal duplication on 16q12.2, a region that includes the *FTO* gene [39].

## *In silico* and *in vitro* analyses of *FTO*

Clues from the Ft mouse and human chromosomal duplication were not sufficient to shed light on the specific effects of *FTO*. Moreover, *FTO* was not annotated in the public databases as having homology to any other known gene. The discovery that this mysterious gene was associated with human obesity [6,7,9] inspired further research aimed at elucidating its functional properties. *In silico* analyses of the human *FTO* sequence revealed homologs in other vertebrates (from fish to mammals) and marine algae (from unicellular photosynthetic picoplankton to multicellular seaweed) [40–43]. Sequence analysis showed that *FTO* shares features with Fe (II) and 2-oxoglutarate (2OG) oxygenases [41,43]. These enzymes catalyze oxidative reactions on multiple substrates using non-heme iron as a co-factor and 2OG as a co-substrate [41,43]. Within this superfamily, FTO is most similar to the *Escherichia coli* enzyme AlkB and its eukaryotic homologs, which can repair DNA methylation damage by hydroxylating methyl groups on the DNA leading to their removal [44]. These data suggest that FTO might act as a demethylase. Sequence analysis also predicted that human FTO and its vertebrate homologs are globular proteins that carry a nuclear localization signal and are unlikely to be targeted to membranes or organelles [42,43]. This prediction was confirmed by *in vitro* studies, which showed that murine Fto is indeed a 2OG oxygenase that can catalyse nucleic acid demethylation [41]. It is conceivable, therefore, that the nucleic acid demethylation activity of FTO might regulate the expression of genes involved in metabolism and that dysregulation of this process might lead to obesity.

## *In vivo* studies of *Fto* in animal models

Caution is always recommended when interpreting gene function from *in vitro* studies, as the activity of molecules might differ in whole organisms and under different conditions: therefore, animal models are used to further elucidate *in vivo* function.

### Wild type Fto expression in fed, fasted and obese rodent models

Early studies of mouse *Fto* and human *FTO* mRNA expression showed that both are ubiquitous, with particularly high levels of expression in the brain and hypothalamus [7,9,34]. These were intriguing results because the hypothalamus is a key site for regulation of energy balance, and genes responsible for monogenic obesity function in the hypothalamus to regulate appetite [45]. Further studies of *Fto* mRNA expression in wild-type rodent tissues confirmed its ubiquitous expression, with high levels of expression in hypothalamic regions known to play important roles in the regulation of energy intake and expenditure, and suggested that expression of Fto might be regulated by nutritional status [40,41]. In the fasted state (that is, when there is a strong stimulus for eating), mice exhibit a significant reduction in hypothalamic *Fto* mRNA expression compared to fed controls. This effect is not rescued by supplementation with the anti-starvation hormone leptin [41,46], which suggests that the reduced hypothalamic *Fto* expression observed during fasting is independent of leptin levels. In support of this conclusion, hypothalamic *Fto* expression is reduced in fasted Lep^ob^ mice (which lack leptin) compared to fed Lep^ob^ and control mice [46]. These studies suggest that *Fto* is downregulated during fasting and upregulated during feeding, and that variation in *Fto* resulting in decreased expression or activity might provide a signal that promotes feeding and obesity.

In contrast to the mouse data, *Fto* expression was increased significantly in the hypothalamus of food-deprived and food-restricted rats [40]. Two possible reasons have been suggested to explain this discrepancy: different sensitivity to starvation in mice and rats, or different times at which samples were taken in the two studies. In rats, in addition to hypothalamic expression, *Fto* was widely and consistently expressed in brain regions related to circadian rhythmicity [40] and therefore its expression might vary at different times of day. Upregulation of hypothalamic *Fto* in fasted rats suggests that high levels of Fto protein might stimulate food intake, which is the opposite effect predicted from its expression in wild type mice [40]. More recent data in rats disagree with this study, finding that over-expression of *Fto* mRNA in the hypothalamus decreased food intake, whereas a 40% decrease in Fto protein led to increased food consumption [47]. These data might contrast with previous data in rats because the measurements and manipulation of *Fto* were limited to a specific region of the hypothalamus (the arcuate nucleus), where the expression of *Fto* is very high. To date, all studies of rodents have been done over prolonged periods of fasting, which might better resemble starvation rather than fasting and hence some of the observations might reflect that. There is a need for studies with shorter fasting times in rodents to better understand the regulation of Fto expression during this time.

Given that variants in *FTO* are associated with human obesity and that *Fto* mRNA levels appear to be regulated in response to feeding and fasting, its expression was determined in relation to changes in obesity. In six mouse models of obesity (A^y^, Lep^ob^, Lepr^db^, Cep^fat^, tub and mice with diet-induced obesity (DIO); Glossary), hypothalamic *Fto* expression did not differ significantly from that in wild type mice [46]. This study assessed *Fto* gene expression in other metabolically relevant tissues, such as fat (adipose) tissue. Adipose tissue acts as a fat store and synthesizes and secretes a variety of proteins (such as leptin) that influence appetite and metabolism at distal sites. Expression of *Fto* in mesenteric fat was reduced significantly compared to wild type in all mouse models except DIO. This is interesting because murine *Fto* gene expression was shown to be downregulated under fasting conditions, suggesting that obese mouse models mimic the fasted state, possibly contributing to their over-eating.

*Fto* expression differs under feeding and fasting conditions and displays tissue-specific differences in mouse models of obesity, but it is not known whether these differences are the cause or the consequence of obesity. To further investigate whether differences in *Fto* expression or function can cause increases or decreases in fat mass, mice harboring *Fto* mutations were generated.

### Mouse models of Fto deficiency

Two mouse models of *Fto* deficiency have been reported: a null mutation (*Fto*^−/−^) resulting in the complete absence of Fto protein expression [48], and a partial loss-of-function mutation with reduced Fto protein levels (Table 1) [49]. The partial loss-of-function *Fto* mouse model carries a point mutation resulting in a change in the amino acid sequence from isoleucine to phenylalanine at position 367 (I367F). Although this residue is outside the catalytic core, it is located within a ∼20 amino acid block conserved throughout vertebrates that defines a new functional domain [49]. *In vitro* experiments demonstrated that, although the full-length I367F protein was correctly localized to the nucleus, it displayed lower levels of expression in mammalian cells and resulted in reduced catalytic activity, possibly through altering the Fto dimerization state. However, the exact role of different Fto dimerization states in energy balance is poorly understood. The mutant Fto I367F protein retains partial function, which is likely to account for some of the observed differences between the phenotypes of these two animal models. Namely, although both models demonstrated reduced body weight and fat mass, this starts early in life in the case of *Fto*^−/−^ mice, whereas weight reduction in *Fto*^I367F^ mice has a maturity-onset. Also, there is no discernible phenotype in the Fto null heterozygous mice, whereas the phenotype of the heterozygous *Fto*^I367F^ is very similar to that of their homozygous mutant littermates. The importance of I367 in dimerization might help explain why the *Fto*^I367F^ heterozygous mice have a phenotype: the I367F substitution might have a dominant negative effect by disrupting the function of wild type Fto through formation of heterodimers. The weight reduction observed in the two models is also very different: *Fto* null mice have a 30–40% weight reduction compared to wild type littermates, whereas the *Fto*^I367F^ mutants have only a 10% reduction in weight. Another significant difference between the two models is that only *Fto*^−/−^ mice exhibited growth retardation and early perinatal death [48,49]. On a high-fat diet, both models exhibited reduced weight gain and reduced white adipose tissue compared to controls [48,49]. These findings indicate that disruption of Fto activity can protect against diet-induced obesity.

These studies then investigated whether reduced growth and adiposity in the *Fto* mouse models is due to decreased energy intake, increased energy expenditure or both. Compared to wild type littermate controls, there was no difference in absolute food intake in *Fto*-deficient mouse models [48,49]. However, *Fto*^−/−^ mice ate relatively more given their reduced body weight and size [48]. These results demonstrated that reduced fat mass in *Fto*-deficient mice was not due to reduced food intake. However, both models exhibited higher levels of energy expenditure (higher metabolic rate) such that overall the animals gained less weight and were protected from obesity [48,49]. The increase in energy expenditure was unrelated to levels of physical activity (indeed, paradoxically there was a reduction in locomotor activity in Fto null mice but this was not observed in the *Fto*^*I*367F^ mutant) but was potentially mediated by increased sympathetic nervous system (SNS) activity, a system that originates in the spinal cord with projections to peripheral tissues and is used by the hypothalamus to regulate energy homeostasis [48,49]. This increased SNS activity might be promoting lipolysis and thermogenesis in adipose tissue and muscle.

Microarray analysis of white adipose tissue, liver and skeletal muscle detected some differences in gene expression in *Fto*^*I*367F^ mutants compared to wild type mice [49]. As expected, given the known link between excess adipose tissue and induction of inflammatory responses, in mutant mice with reduced white adipose tissue mass, expression of multiple genes involved in inflammation was downregulated in adipose tissue. In contrast, upregulation in adipose tissue of some genes involved in fatty acid catabolism might explain, in part, the lower fat mass. Upregulation of genes involved in fatty acid synthesis in adipose tissue and upregulation of fatty acid synthase and genes involved in carbohydrate metabolism in muscle might reflect secondary adaptations to a lower supply of fatty acid (due to smaller fat reserves). Indeed, *Fto*^I367F^ mutants demonstrated increased carbohydrate metabolism relative to fat metabolism. Hypothalamic neuropeptide expression did not differ between wild type and mutant in the fasted state, but Npy expression was lower in fed mutant mice. Given that increased Npy stimulates food intake, this finding suggests that Fto-deficient mice are more sensitive to satiety and are thereby protected from obesity through over-eating. A blunting of Npy induction was seen in *Fto*^−/−^ mice in the fasted state, lending support to the suggestion that Fto promotes Npy expression [48]. It is conceivable that the nucleic acid demethylation activity of Fto might provide a mechanism through which it affects expression of these other genes.

The phenotypic characterization of both the *Fto*^−/−^ and *Fto*^I367F^ mice support the idea that association of *FTO* SNPs with human obesity arises via regulatory or functional effects on *FTO* rather than other genes in the region. Although the *Fto* null mutation could impact regulation of other genes, the non-synonymous mutation in *Fto*^I367F^ mice is most likely to exert its functional effects on *Fto* alone [49]. Furthermore, both models suggest that alleles associated with increased risk of obesity will cause up- or dysregulation of *FTO* and that inhibition of *FTO* might protect against obesity [48,49].

## The relevance and utility of functional *FTO* data for humans

Although animal models are useful tools to investigate gene function, they do not necessarily reflect the situation in humans. Recently, for example, a loss-of-function non-synonymous mutation resulting in an arginine to glutamine change at position 316 in *FTO* was reported to segregate with an autosomal recessive disease in a large Palestinian Arab consanguineous family with nine affected members [50]. The syndrome includes post-natal growth retardation, head and face dysmorphism, severe psychomotor delay, functional brain deficits and, in some patients, brain malformations, cardiac defects, genital abnormalities and cleft palate. These developmental characteristics seem more similar to the Ft mouse than either of the *Fto* mouse models. This discrepancy between the effects of human and mouse mutations (Table 1) might reflect differences between the actions of *FTO* in humans and mice or, alternatively, multiple roles for *FTO* that are disparately affected by the mouse and human mutations. This is an intriguing area of investigation for the future and results from different animal models (e.g. transgenic *Fto* over-expression in mice) are eagerly anticipated. The lack of clinical obesity in patients carrying R316Q and family members is perhaps consistent with the relationship between loss of *Fto* function and leanness in mice. More recently, however, loss-of-function heterozygous mutations in *FTO* were found in both lean and obese humans, suggesting that haploinsufficiency does not protect against obesity completely, though the relationship might be complicated by other obesity-promoting factors [51]. Other differences between the effects of mouse *Fto* mutations and human *FTO* SNPs (Table 1) are perhaps less surprising because *FTO* SNPs in the human population are likely not strong loss-of-function variants, particularly as the most strongly associated SNPs are intronic and therefore postulated to have an effect on gene regulation.

## Concluding remarks and future perspectives

Despite recent progress, the mechanism by which SNPs in *FTO* influence human body mass remains elusive. Multiple processes could plausibly contribute to the risk of obesity, including neurological circuits governing appetite and whole-body energy expenditure, as well as peripheral pathways involved in energy expenditure. Loss-of-Fto function appears to reduce fat mass in mice, at least in part, through increased energy expenditure but not decreased energy intake [48,49]. However, the study of intermediate phenotypes in humans showed that *FTO* SNPs are associated with appetite and food intake but not energy expenditure (Table 1). Interestingly, data from rodents suggested that *Fto* might affect neuropeptide Y expression in the hypothalamus, which in turn is known to impact feeding behavior. An investigation of the association between *FTO* SNPs/expression and neuropeptide levels in human hypothalamus might therefore provide a mechanism for the modulatory effect of *FTO* SNPs on appetite, although it would be challenging to obtain human hypothalamic material. Another potential area of future investigation is the role of *FTO* in circadian rhythms, given its expression in relevant brain regions in rats [40]. Aberrant circadian rhythms have been linked to metabolic disease and obesity [52].

At present, the strongest associations between *FTO* SNPs and BMI belong to intronic SNPs, which might have a role in the regulation of *FTO* and/or nearby genes. It is important to note, however, that associated SNPs are not necessarily the causal SNPs underlying the association. It is possible that fine-mapping the causal variant(s) could shed light on the biological mechanism impacting body mass, for example by determining whether the causal variant(s) change the amino acid sequence of FTO or whether they lie within a control element affecting expression of other genes in the region, such as *RPGRIP1L*. However, fine-mapping the association signal might be difficult because the obesity-associated SNPs lie within a 47 kb LD block in which the effects of causal variant(s) could be indistinguishable from other, highly correlated proxies. Under these circumstances it might prove more important to understand the biological effect of the risk haplotype (rather than the causal variants themselves) on genes and pathways. *FTO* obesity-associated SNPs do not appear to influence *FTO* expression but could be investigated for their effect on expression of its neighboring gene *RPGRIP1L*. Nevertheless, there is now some evidence to suggest that the associations with BMI are indeed mediated through *FTO* (Box 2); however, joint effects via *FTO* and *RPGRIP1L,* or other genes, have not been dismissed completely. A recent report suggested that the obesity-associated *FTO* region contains highly conserved non-coding elements that might be controlling expression of *IRX3* (encoding a transcription factor important in development). Though none of the obesity-associated SNPs fall within these elements, the possibility that the association signal results from correlation with causal variant(s) within these regions has not been excluded [53].

It is clear that the journey undertaken by *FTO* has generated many questions as well as answers. Perhaps the most important lesson from the extensive work on *FTO* over the last three years is the promise that other obesity loci identified by GWAS could lead to new understanding of the underlying biology of human adiposity. Despite the small effect sizes and predictive value of the common variants identified to date, these hits often represent previously unsuspected obesity genes. The story of *FTO* research provides valuable lessons for the study of other obesity-associated genes. Ultimately, it is hoped that knowledge of susceptibility gene function will highlight novel genes and molecules that could be targeted for therapeutic intervention. For example, the data presented in this review indicate that *FTO* deficiency protects against obesity. However, the devastating effects of a loss-of-function non-synonymous mutation in *FTO* in a human family emphasizes that care should be taken in developing drugs to alleviate obesity by reducing the expression or inactivating catalytic activity of *FTO* [50]. Clinical and translational effects should not be expected in the near future but GWAS and subsequent investigation of obesity genes promises exciting new insights into common obesity in the next few years.

**Glossary**

**A**^**y**^**:** the lethal yellow (A^y^) mutation in the agouti gene produces mice with a complex phenotype including obesity and a yellow coat color [54].
**Body mass index (BMI):** weight (in kilograms) divided by the square of height (in meters). Overweight is defined as BMI >25, and obesity as BMI >30.
**Cep**^**fat**^**:** homozygous loss-of-function mutations in the carboxypeptidase E gene result in obesity and hyperglycemia [55].
**Diet-induced obesity (DIO):** mice with obesity induced by diet, rather than purely genetic causes [56].
**Dominant negative:** a mutation that results in a protein that interferes with the function of the wild type protein, usually through dimerization with the wild type protein.
**Energy balance:** energy intake versus energy expended as internally produced heat and physical activity.
**Lep**^**ob**^**:** disruption of both copies of the leptin gene results in obese, hyperphagic (over-eating) mice [57].
**Lepr**^**db**^**:** shares similar features with the Lep^ob^ mouse and results from abnormal splicing of the long, hypothalamic leptin receptor [58].
**Linkage disequilibrium (LD) block:** linkage disequilibrium is the name given to the phenomenon whereby alleles at multiple loci appear together more often than would be expected by chance. This is often measured by *r*^2^, which ranges from zero (unlinked loci) to 1 (complete correlation between loci). An LD block defines a region within which variable loci are highly correlated (usually r^2^ >0.8).
**Lipolysis:** the breakdown of fat (triglycerides) into free fatty acids within body cells.
**Polydactyly:** having >10 fingers or toes.
**Tagging SNPs:** SNPs that can be used as proxy for other, correlated SNPs (normally with a pairwise correlation of *r*^2^ >0.8 (see the definition of linkage disequilibrium block, above)).
**Thermogenesis:** heat production. A component of energy expenditure that can be stimulated by the sympathetic nervous system.
**Tub:** tubby mice exhibit adult-onset obesity due to mutation in the Tub gene [59,60]. The function of the Tub gene is not entirely clear.

## Figures and Tables

**Table 1:** Comparison of the effects of *FTO* variants in mouse and human

Theme	*Fto*^−/−^ mouse*	*Fto*^I367F^ mouse**	*FTO* R316Q in human*	*FTO* variation in human	References
Pre- and post-natal body weight	No effect on pre-natal development but decreased body weight is apparent from an early age, with reduction in fat mass more pronounced than reduction in lean mass. Reduced weight gain on high-fat diet	No effect on pre-natal development but males exhibit maturity-onset reduction in body weight, attributable to decreased fat mass. Reduced weight gain on high-fat diet	Parents of the probands are not clinically obese, nor reported to be excessively thin	Robust association between *FTO* SNPs and fat mass in children, and a trend towards increased birth weight and mass in relation to height in newborns carrying obesity risk alleles, though this was statistically significant in only one study	[4,7,9–11,15,18,48–50,66–72]

Post-natal death	Post-natal death occurred more frequently	No difference in post-natal mortality	Death from intercurrent infection or an unidentified cause occurred within 30 months of age	Association with post-natal mortality but less associated with disease incidence, implying a reduced ability to cope with disease in risk allele carriers	[48–50,73]

Growth retardation	Growth retardation from post-natal day 2	No growth retardation	All affected individuals suffered from post-natal growth retardation	No association with height	[7,48–50,74]

Development	No other gross developmental abnormality	No other gross developmental abnormality	Microcephaly, severe psychomotor delay, functional brain deficits, and facial dysmorphism. Structural brain malformations, cardiac defects, genital anomalies, and cleft palate observed in some patients	No difference reported	[48–50]

Adipose tissue mass and adipokines	Decrease in adipose tissue mass, leptin and increase in adiponectin	Decrease in adipose tissue mass. Higher leptin secretion per unit of body fat. No difference in adiponectin	No difference reported	Association driven by general changes in fat mass, not lean mass. No convincing association with leptin or adiponectin	[7,10,11,16,22,48–50,75–77]

*FTO* expression and function	Abolished expression in all tissues tested	Reduced expression in mammalian cells, and disrupted dimerization and catalytic activity of Fto	FTO R316Q is catalytically inactive. This amino acid substitution does not affect FTO nuclear localization	No reported association between *FTO* SNPs and FTO expression. However, there is some evidence that *FTO* expression is enhanced in adipose tissue of obese individuals. No reported effects on catalytic activity	[27,48–50,77–80]

Sex differences	Reduced body weight more pronounced in males, but amongst heterozygotes only females showed reduced body weight at 20 weeks	Only male Fto^I367F^ mice exhibited reduced weight at 12 weeks	No difference reported	Overwhelmingly no evidence for gender difference in the effect of *FTO* SNPs, despite hints of a stronger effect in girls	[7,9,48–50,66]

Energy intake	Higher food intake in *Fto*^−/−^ mice relative to lean mass	No difference in food intake between *Fto*^I367F^ mutant and control mice	No difference reported	Some studies report no association with food intake, though in other studies (especially large studies with children where diet is reported by parents or through feeding experiments) there have been statistical associations with increased energy intake or preference for energy dense foods, and one report of an interaction with *FTO* genotype on BMI	[11,13,48–50,81–91]

Energy expenditure	Increased energy expenditure	Increased energy expenditure	No difference reported	No association with measures of energy expenditure. No correlation between skeletal muscle or adipose tissue *FTO* expression and energy expenditure	[11,13,48–50,78,81,84,92,93]

Physical activity	Significantly decreased physical activity	No difference in physical activity	No difference reported	No association with physical activity, but reported interaction between genotype and physical activity on BMI	[10,25,48–50,67,82,90,91,94,95]

Glucose tolerance and insulin sensitivity	Mild improvement in insulin sensitivity (probably as a consequence of leanness)	No convincing difference in glucose tolerance or insulin sensitivity	No difference reported	Overwhelmingly the evidence suggests no association with glucose tolerance or insulin sensitivity of *FTO* SNPs, or *FTO* expression	[12,14,16,20–24,48–50,71,78,80,96]

Lipids		Triglycerides and high-density lipoprotein (HDL) cholesterol increased in *Fto*^I367F^ mice	No difference reported	Some evidence for association with elevated triglycerides and cholesterol	[17,48–50,80,82,96]

Other gene expression	*Npy* mRNA induction was blunted and *Pomc* mRNA repression exaggerated in fasted *Fto*^−/−^ mice	Altered expression of some genes involved in inflammation, fatty acid catabolism and synthesis, carbohydrate metabolism and the ER stress response in *Fto*^I367F^ mice and *Npy* expression was lower in fed mutant mice	No difference reported	Not examined for *FTO* SNPs, but *FTO* expression correlated with oxidative phosphorylation genes involved in mitochondrial function and their regulator, *PGC1A*, as well as *GLUT4* mRNA. Some evidence for a role of *FTO* in inflammation and stress response	[6,48–50,78,97]

* Cells refer to the homozygous mutant unless otherwise stated.

** Cells refer to both the homozygous mutant and heterozygous mice unless otherwise stated.

## Box 1 Fine-mapping association between FTO and obesity

Whereas linkage disequilibrium (LD) aids the discovery of genetic associations, because the investigator does not need to test all variant(s) in a given genomic region to detect association signals, it is a hindrance to fine-mapping efforts. This is because high correlation between SNPs makes it difficult to distinguish between the effects of true causal variants and correlated non-causal variants. Consequently, it is not clear which causal variant(s) underlie the association between BMI and SNPs in *FTO* (which reside within a 47 kb LD block). So far, fine-mapping attempts have focused on selecting tagging SNPs within the associated genomic region and genotyping or imputing these in additional study populations of different ancestry [6,9,15,19,25,26]. The advantage of using study cohorts of different ancestry lies in their varying patterns of LD. For example, LD is generally weaker in populations of African descent. Two recent studies attempted to refine the association signal from *FTO* in individuals of African ancestry, but none of the SNPs tested was associated with obesity in both cohorts [6,15]. Fine-mapping studies have been done with Japanese [19] and Old Amish Order participants [25]. Some of the SNPs associated with BMI in these populations have overlapped with associated SNPs in Europeans but the SNPs most strongly associated with BMI vary between cohorts. For example, the strongest SNP (rs1861868) in the Old Amish Order study had not been previously associated with obesity. This might reflect interstudy differences in the causal variant(s) or differences in levels of LD between tested SNPs and the same causal variant(s). It is worth mentioning that in a European fine-mapping study cohort, three of the four most significantly associated SNPs (rs17817449, rs3751812 and rs1421085) were in putative functional regions (based on conservation and predicted impact on regulatory domains) but these SNPs were highly correlated with each other and their effects could not be distinguished [9]. Interestingly, despite an earlier demonstration that associated SNPs in the *FTO* region were likely to be tagging the same signal rather than multiple, independent signals [6], Tonjes *et al*. discovered an independent association with BMI from SNPs in *FTO* introns 2 and 3, downstream of the original signal, in Sorbian individuals [26]. This suggests that this genomic region might harbor multiple variants that influence susceptibility to obesity, which might complicate future fine-mapping attempts.

## Box 2 FTO or RPGRIP1L?

Given the close proximity between *FTO* and *RPGRIP1L*, it is possible that SNPs in *FTO* are associated with obesity through a direct regulatory effect on *RPGRIP1L* or an indirect effect (through linkage disequilibrium with *RPGRIP1L* or with *RPGRIP1L* regulatory site variation) on *RPGRIP1L* function. For example, the obesity-associated *FTO* SNP, rs8050136, lies within a Cut-like 1 (CUTL1) transcription factor binding site. CUTL1 preferentially binds the rs8050136 allele associated with protection against obesity [46]. Short interfering (si) RNA-mediated knockdown of CUTL1 in fibroblasts decreased both *FTO* and *RPGRIP1L* expression [46]. RPGRIP1L is located in the basal body of cilia, where it regulates sonic hedgehog (SHH) signaling [61]. Ciliary defects have been associated with obesity in mice and humans [62,63]; indeed, the disruption of genes involved in cilia formation or maintenance leads to obesity [62,63]. It is therefore possible that human obesity is influenced by *FTO* SNPs via an effect on *RPGRIP1L* expression and ciliary function. However, several pieces of evidence suggest that this association is driven by a direct effect on *FTO*:

(1) Mice homozygous for a targeted mutation in *Rpgrip1l* have a phenotype reminiscent of the Ft mouse [34], with head and facial abnormalities, disruption of CNS development, disturbances of left–right asymmetry, and polydactyly [61]; but no difference in adiposity has been reported. In contrast, mouse models of *Fto* deficiency exhibit reduced fat mass and body weight [48,49].
(2) Mutations in human *RPGRIP1L* result in ciliopathies, Joubert syndrome (JBTS) and Meckel syndrome (MKS) [64,65], but no difference in adiposity compared to the general population has been reported.
(3) Four known *RPGRIP1L* SNPs lack an association with either obesity or related traits [66].
(4) *Rpgrip1l* mRNA expression is normal in an *Fto*-deficient mouse model which exhibits reduced body weight and fat mass [48]
(5) Fasting is associated with decreased *Fto*, but not *Rpgrip1l*, expression in mesenteric fat in mice [46].
